# Supplementary material for: Multifunctional and biodegradable self-propelled protein motors
Source: Nat Commun. 2019 Jul 18;10:3188. doi: 10.1038/s41467-019-11141-9 (PMC6639312; doi:10.1038/s41467-019-11141-9)
Supplement: Supplementary file 1 — Supplementary Information [file 41467_2019_11141_MOESM1_ESM.pdf]

Multifunctional and Biodegradable Self-Propelled Protein Motors

Pena-Francesch et al.

SUPPLEMENTARY INFORMATION

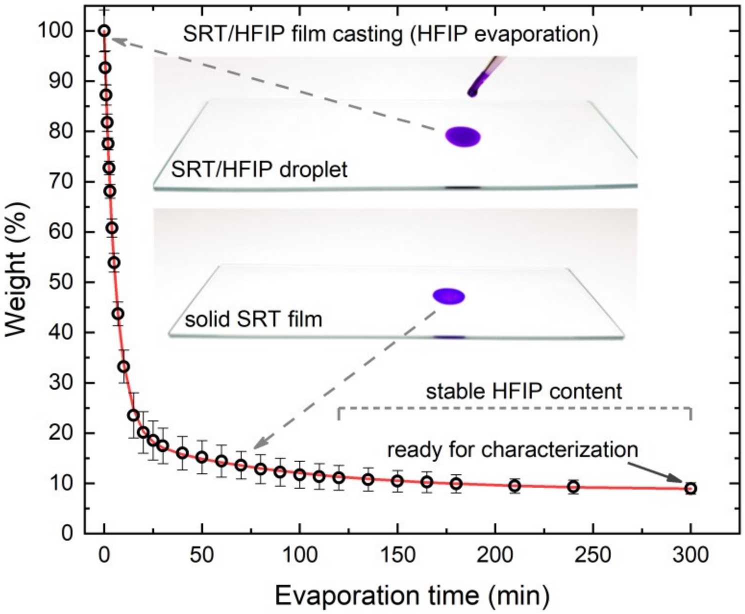

Supplementary Figure 1. Thin film casting of SRT protein from HFIP solutions. During the first 30 minutes, the majority of HFIP solvent evaporates and SRT protein begins self-assembling into a solid hydrogen-bonded network. After two hours, solvent evaporation stabilizes and the protein film is ready for fabrication and characterization. Error bars represent standard deviation, n=5.

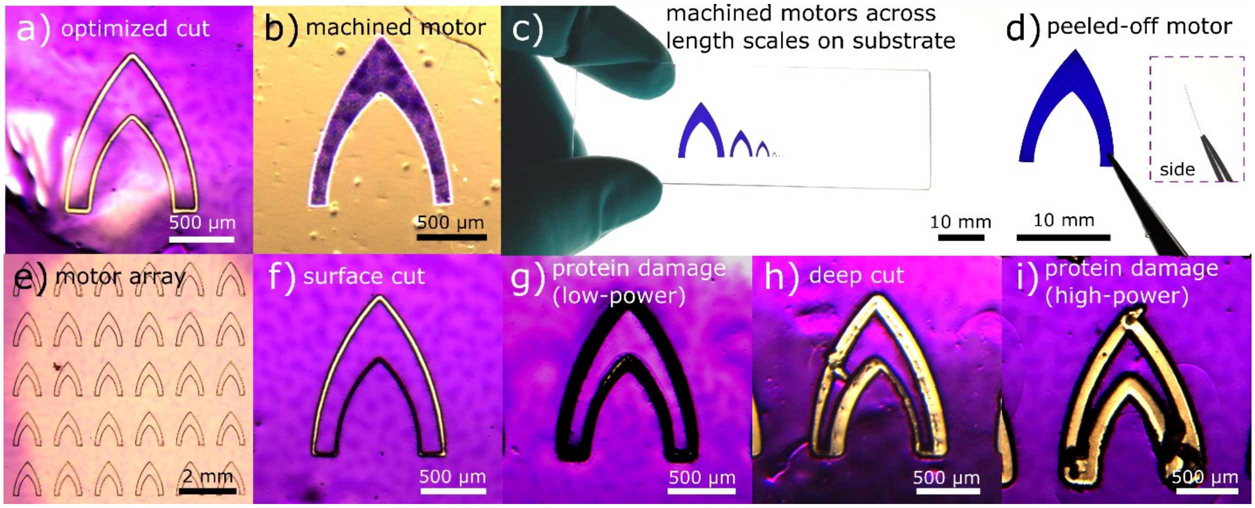

Supplementary Figure 2. **Laser micromachining of protein motors.** a) Protein motor machined with optimized process parameters. b) Protein motor after peeling off surrounding film. c) Machined motors across length scales (from 10 mm down to 100 µm) on a soft PDMS substrate (same motors as **Figure 1b**). d) Motor (l = 10 mm) peeled off the substrate with tweezers without deformation. e) 6x5 array of micromachined l = 1 mm motors. f) Defective surface cut due to low cycle repetitions. g) Low-power protein damage due to short cycle delay. h) Deep cut into the substrate due to high cycle repetitions at mid power. i) Protein damage due to high-power machining.

Protein films were micromachined with a LPKF ProtoLaser U3 scanner-guided UV laser (wavelength 355 nm) with 189 mW power at 50 kHz frequency for 50 cycles with 1500 ms delay. Using these optimized parameters, the protein films were cut following the specified CAD model with no visible defects (**Supplementary Figure 2a**). Next, the surrounding protein film was peeled off, leaving the machined motors on the soft PDMS substrates (**Supplementary Figure 2b**, **Supplementary Figure 2c**). Then, the machined motors were individually peeled off the substrate with tweezers (**Supplementary Figure 2d**), preserving their geometry and mechanical integrity due to their good mechanical properties. The motors were then transferred for swimming experiments and characterization. This method was used to fabricate protein motors with characteristic length scales *l* ranging from 10 mm down to 100 µm, and it can successfully fabricate large arrays of motors simultaneously (**Supplementary Figure 2e**). Each motor was individually inspected in a microscope, and those with visible fabrication defects (protein impurities, microbubbles, heterogeneities, defects from laser cutting) were discarded (approximately 5% from the total fabrication).

Laser machining with the improper parameters prior to process optimization resulted in defective motors:

- Surface cut (**Supplementary Figure 2f**): machining at low power (189 mW) with low cycle repetition resulted in surface cutting only instead of cutting through the full 20 µm thickness of the films. Since the motors remained connected to the surrounding film through uncut regions, they were peeled off the substrate together with the film. A minimum of 50 cycle repetitions were determined as optimum parameter.
- Protein damage (low-power) (**Supplementary Figure 2g**): machining at low power (189 mW) with small tool delay (i.e., delay between consecutive cycle repetitions) resulted in protein damage. Continuous consecutive cycles did not allow for heat dissipation and thermally degraded the protein

(black carbonized region). A cycle delay of 1500 ms was enough to avoid thermal degradation in multiple low-power consecutive cycles.

- Deep cut (**Supplementary Figure 2h**): multiple consecutive cycles (50) at mid-power (1 W) with 1500 ms tool delay cut past the protein film into the PDMS soft substrate, complicating the peeling off of the motors. In addition, it resulted in loss of resolution and geometrical defects.
- Protein damage (high-power) (**Supplementary Figure 2i**): a single cycle at high power (5 W) damaged the protein film via thermal degradation, resulting in severe geometrical defects and loss of mechanical integrity.

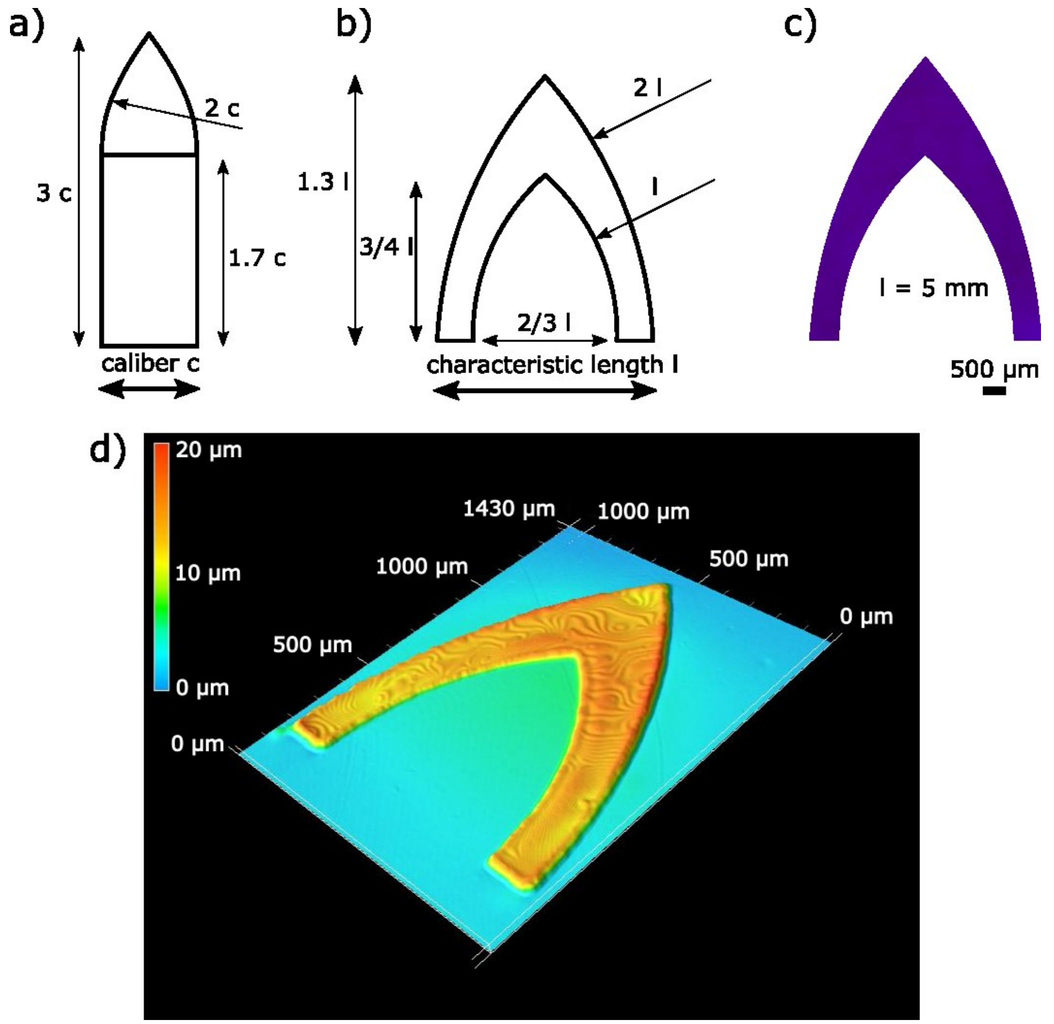

Supplementary Figure 3. Protein motor design parameters. a) Design parameters of G1 ballistic coefficient profile (standard in ballistics) as function of caliber *c*. b) Bullet-inspired design parameters of the protein motors as function of characteristic length *l* (analogous to caliber *c*), with a posterior cavity to generate an asymmetric surface tension gradient. c) Stereomicroscopy image of a *l* = 3 mm laser-cut protein motor. d) 3D laser map of a *l* = 1 mm protein motor, with a thickness of 20 µm. Color scale is height from 0 to 20 µm.

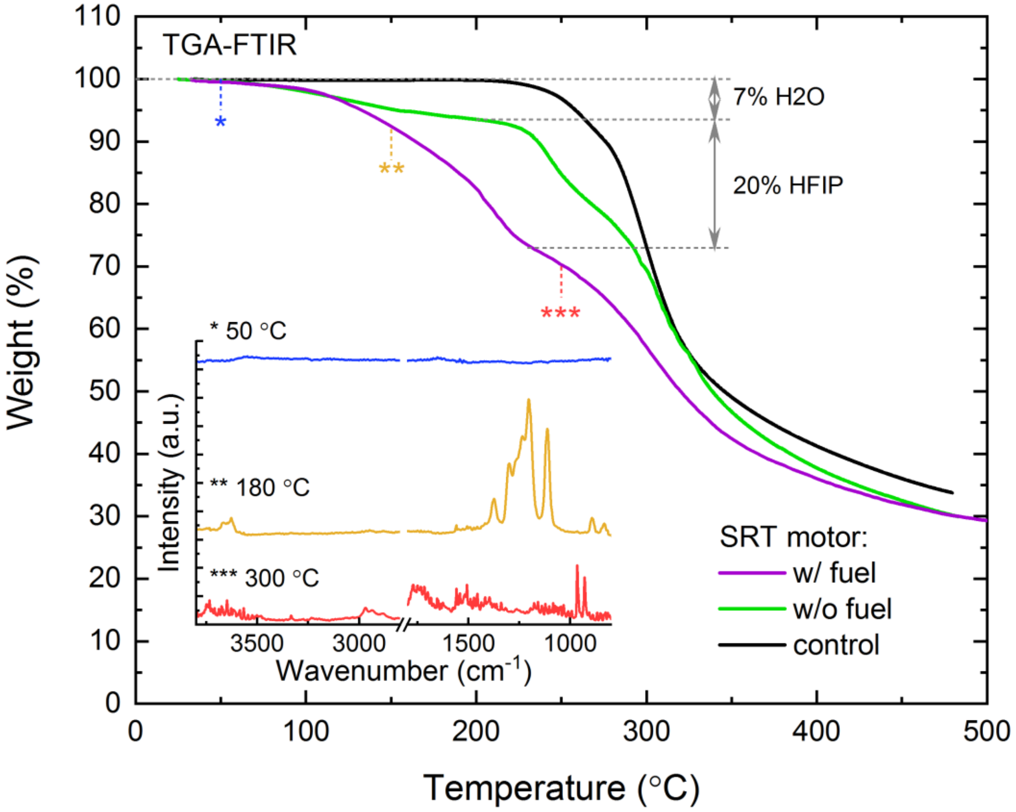

Supplementary Figure 4. Thermogravimetric analysis coupled with infrared spectroscopy of SRT materials.

As-cast (w/ fuel) SRT films exhibit three weight loss events:

(*) 50 – 120 °C: evaporation of trapped water from ambient humidity. FTIR spectrum shows small bands at 3500 cm^{-1} and 1700 cm^{-1} corresponding to H_2O.

(**) 120 – 240 °C: evaporation of trapped HFIP. FTIR spectrum shows absorption bands in the 1100-1300 cm^{-1} range corresponding to HFIP.

(***) 240 – 500 °C: thermal degradation of the protein. FTIR spectrum shows noisy absorption bands in the 800-1800 cm^{-1} range corresponding to short hydrocarbons as degradation byproducts.

For quantification purposes, "exhausted" (w/o fuel) and "control" (annealed, without humidity absorption) protein samples have been analyzed, revealing a water content of 7 ± 1% and HFIP content of 20 ± 1%.

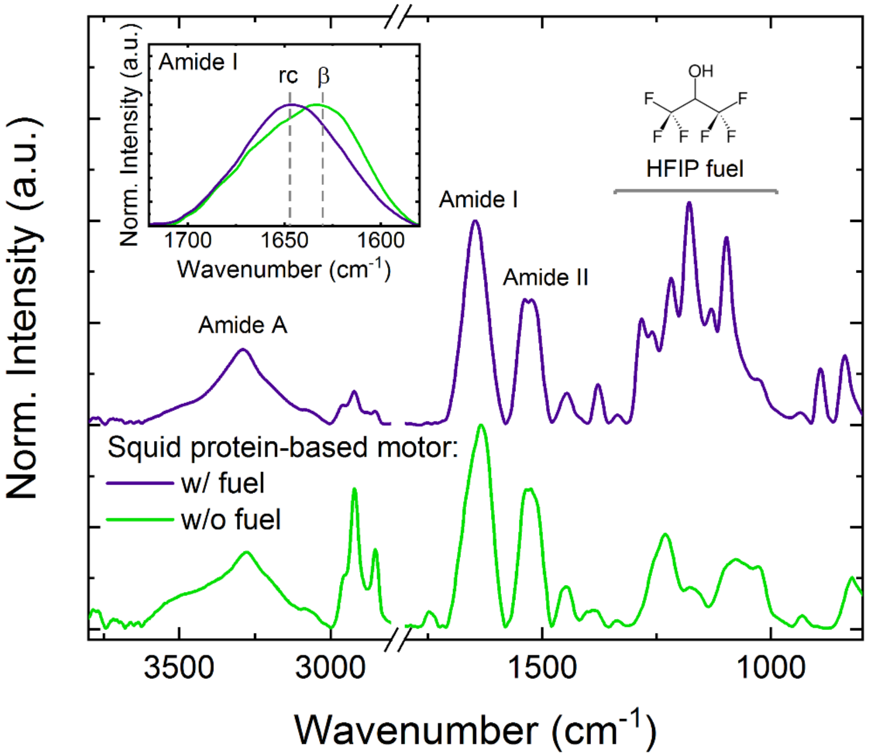

Supplementary Figure 5. Infrared spectroscopy of protein motors with and without fuel reveals formation of β-sheet nanostructures with the release of fuel. Fourier transform infrared (FTIR) spectra of SRT motors (with HFIP fuel and after its exhaustion) reveal typical absorption bands in proteins (amide I, amide II, amide A). High intensity bands corresponding to trapped HFIP appear in the 1000-1300 cm^{-1} region (not visible after fuel exhaustion). The amide I band (carbonyl stretching vibration) shifts from 1650 to 1630 cm^{-1} with the exhaustion of fuel, indication a transition from random coil (rc) conformations to β-sheet (β) nanostructures[1].

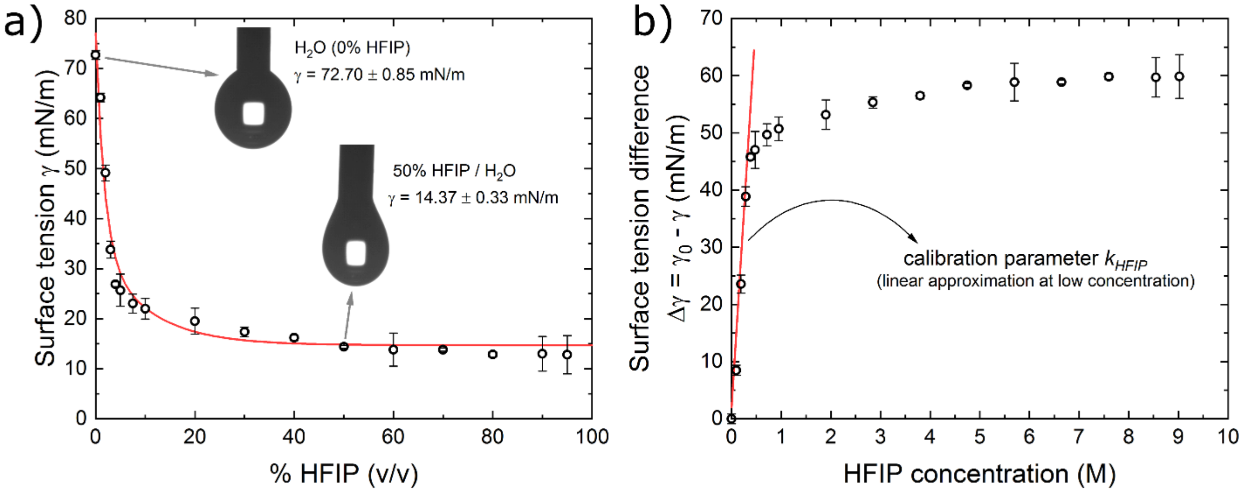

Supplementary Figure 6: Surface tension characterization of hexafluoroisopropanol (HFIP) fuel. a) Surface tension measured in a goniometer by the pendant drop method as function of HFIP concentration (% v/v). b) Difference in surface tension $\Delta\gamma = \gamma_0 - \gamma$ between water media and local HFIP concentration. Error bars represent standard deviation, n=5.

The surface tension of hexafluoroisopropanol (HFIP) fuel was optically measured in a goniometer by the pendant drop method, and it was calculated from analysis of the drop shape using the Young-Laplace equation. We measured the surface tension of HFIP in water in concentrations from 0% to 100% in volume, with surface tension ranging from 72.7 to 14.7 mN/m. This allowed us to estimate the local difference in surface tension when HFIP is released from the motor. We measured the difference in surface tension $\Delta\gamma = \gamma_0 - \gamma$ as function of HFIP concentration in the solution (where γ_0 is the surface tension of the water media, and γ is the surface tension of the HFIP solution). For later calculation and modeling purposes, a motor releasing HFIP can be considered a moving source, and therefore we can assume that the local concentration of HFIP in the close surroundings of the motor will be very low (contrary to a stationary case). Hence, we can make a linear approximation at low HFIP concentrations to $\Delta\gamma$, providing the calibration parameter k_{HFIP} (used in the model described in **Supplementary Note 1**).

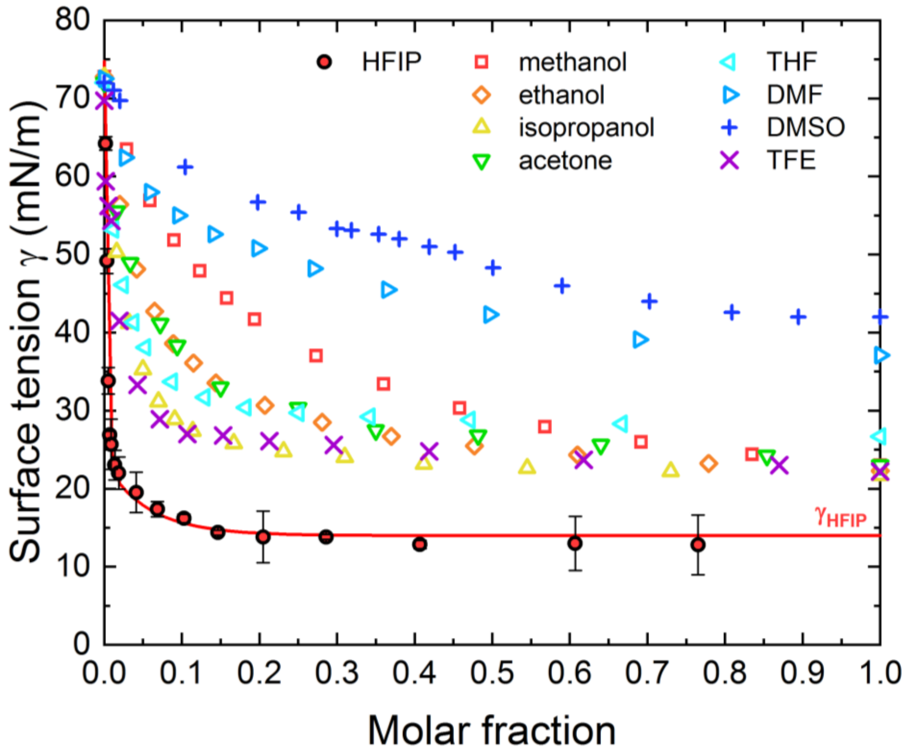

Supplementary Figure 7: Surface tension of chemical fuels in water media as function of fuel molar fraction. Methanol[2], ethanol[2], isopropanol[2], acetone[3], tetrahydrofuran (THF)[4], dimethylformamide (DMF)[5], dimethylsulfoxide (DMSO)[6], and trifluoroethanol (TFE)[7] data taken from their respective bibliography references. Error bars represent standard deviation, n=5.

In **Supplementary Figure 7**, we compare the surface tension of diverse chemical fuels (commonly used in self-propelled systems) in water as function of fuel molar fraction. We can observe that HFIP is advantageous over other chemical fuels for two main reasons: surface tension of pure HFIP (14.7 mN/m) is significantly lower than that of other fuels, and the surface tension at very low concentrations of HFIP is significantly lower than that of other fuels at equivalent concentrations. This low surface tension at very low concentrations has an important implication in the motor locomotion: small amounts of released HFIP fuel (very low local concentrations) will create large surface tension gradients in water, generating stronger Marangoni forces than other fuels at equivalent concentrations. In other words, smaller amounts of HFIP fuel are necessary to generate larger Marangoni forces than other fuels. This translates into very favorable motor metrics (performance output and efficiency), reported in **Figure 4b** and **Supplementary Table 4**.

Supplementary Table 1: Chemical fuels in SRT protein disk motors of Ø = 5mm. Density, viscosity, surface tension, capability of dissolving SRT proteins, and motor mobility lifetime.

Chemical fuel in SRT protein motors	Density (g/cm^3)	Viscosity (cP)	Surface tension (mN/m)	Dissolves SRT	Mobility lifetime (s)
Methanol	0.792	0.69	22.1	✗	28 ± 13
Ethanol	0.789	1.36	33.0	✗	27 ± 21
Isopropanol (IPA)	0.786	0.60	22.3	✗	16 ± 2
Acetone	0.784	0.39	23.7	✗	51 ± 22
Tetrahydrofuran (THF)	0.889	0.52	26.7	✗	10 ± 7
Dimethylformamide (DMF)	0.944	0.79	37.1	✗	199 ± 73
Chloroform	1.490	0.59	27.2	✗	3 ± 2
Dichloromethane (DCM)	1.330	0.43	26.5	✗	3 ± 2
Hexane	0.655	0.30	18.4	✗	6 ± 5
Hexadecane	0.770	2.38	27.5	✗	7 ± 6
Dimethylsulfoxide (DMSO)	1.100	2.00	44.0	✓	171 ± 27
Trifluoroethanol (TFE)	1.325	1.19	22.2	✗	309 ± 169
**Hexafluoroisopropanol (HFIP)**	**1.596**	**1.65**	**14.7**	✓	**2140 ± 92 (steady) (> 2 hours total)**

Another important property of HFIP over other chemical fuels is its capability of dissolving SRT proteins (and other aggregated proteins). Most organic solvents do not dissolve or swell the protein, and therefore the amount of fuel absorbed by the protein is very low (resulting in very short mobility lifetimes in the order of a few seconds). However, HFIP can dissolve SRT proteins to high concentrations, allowing for the facile fabrication of protein motors by film casting and laser micromachining. HFIP fuel is entrapped in the protein matrix (approximately 20%) and slowly released to the swimming media through a β-sheet nanocrystal network. The entrapment of fuel and its slow release result in very long mobility lifetimes compared to other chemical fuels. Disk protein motors (20 μm in thickness and 5 mm in diameter) with HFIP fuel exhibited 36 ± 2 minutes of steady uninterrupted locomotion. After this continuous locomotion regime, the motors exhibit intermittent locomotion for more than 2 hours. This irregular locomotion is caused by the exhaustion of fuel at the motor edges and the internal diffusion and replenishment of fuel from the motor core to the edges[8]. Regardless of whether one is interested in the steady mobility regime (as it is in our case) or in the total mobility regime, the performance of the SRT/HFIP motors is superior compared to other chemical fuels.

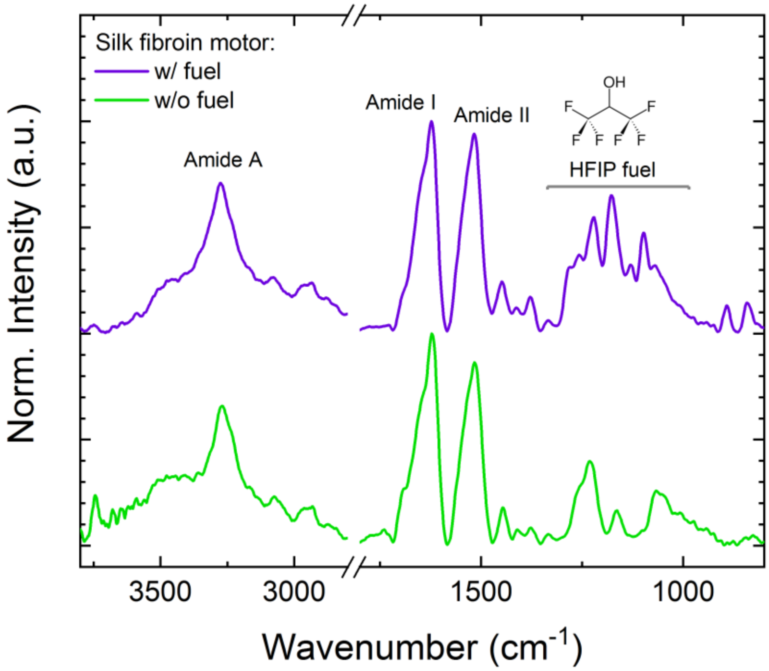

Supplementary Figure 8. Infrared spectroscopy of silk fibroin films. Infrared spectra show typical absorption bands for proteins (amide I, amide II, amide A), but also absorption bands corresponding to trapped HFIP in the 1000-1300 cm^{-1} region. HFIP bands are not visible after the fuel has been exhausted. Amide I bands are centered around 1630 cm^{-1}, characteristic of β-sheet nanostructures, and agreeing with previous reports of HFIP-processed insoluble silk fibroin[9]. Unlike SRT (**Supplementary Figure 5**), the amide I band of fueled and fuel-exhausted silk fibroin films does not shift, indicating that the protein nanostructure (β-sheet network) remains unaffected by the fuel release.

Silk fibroin is a protein similar to SRT in both amino acid sequence (alternating A-rich and G-rich domains) and nanostructure (β-sheet-stabilized semicrystalline network)[10]. However, silk/HFIP motors did not exhibit significant locomotion in water (**Supplementary Movie 1**). We attribute the different behavior to the lack of conformational changes in silk upon exposure to water in analogous conditions to SRT.

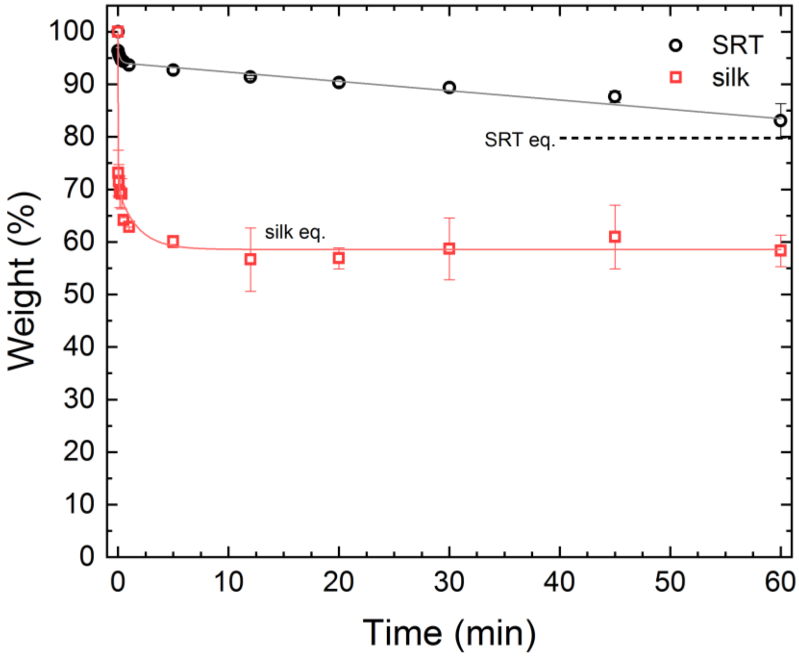

Supplementary Figure 9. Fuel release profile of SRT and silk fibroin proteins in contact with water.

Absorbed water was removed by annealing for every measurement point (only protein + fuel was measured). Silk fibroin films showed a major weight loss during the first minute, indicating a fast release of HFIP (single exponential decay). On the other hand, SRT protein showed a much slower release of HFIP (and longer mobility lifetime) due to the surface release and the internal diffusion through the evolving protein nanostructure (double exponential decay). Error bars represent standard deviation, n=5.

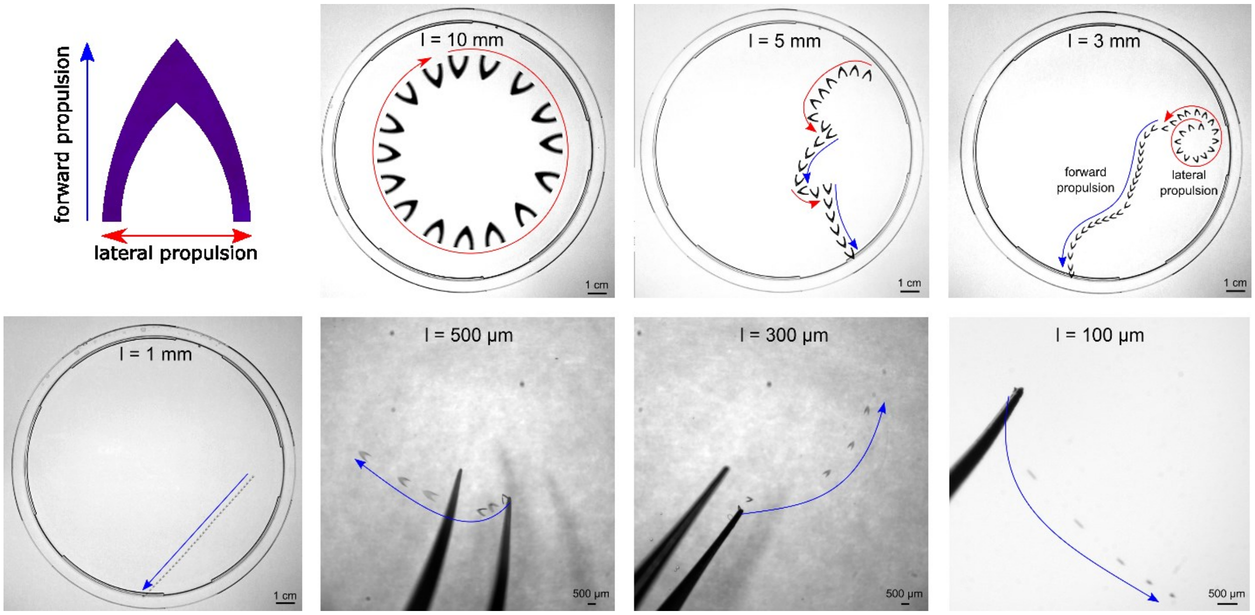

Supplementary Figure 10. Propulsion modes. Locomotion of protein motors ranging from 100 µm to 10 mm. *l* = 10 mm motors exhibit lateral propulsion (red arrow, perpendicular to the leg direction). *l* = 100 µm – 1 mm motors exhibit forward propulsion (blue arrow, along the tip direction). *l* = 3 - 5 mm motors exhibit combined propulsion modes (alternating forward and lateral propulsion).

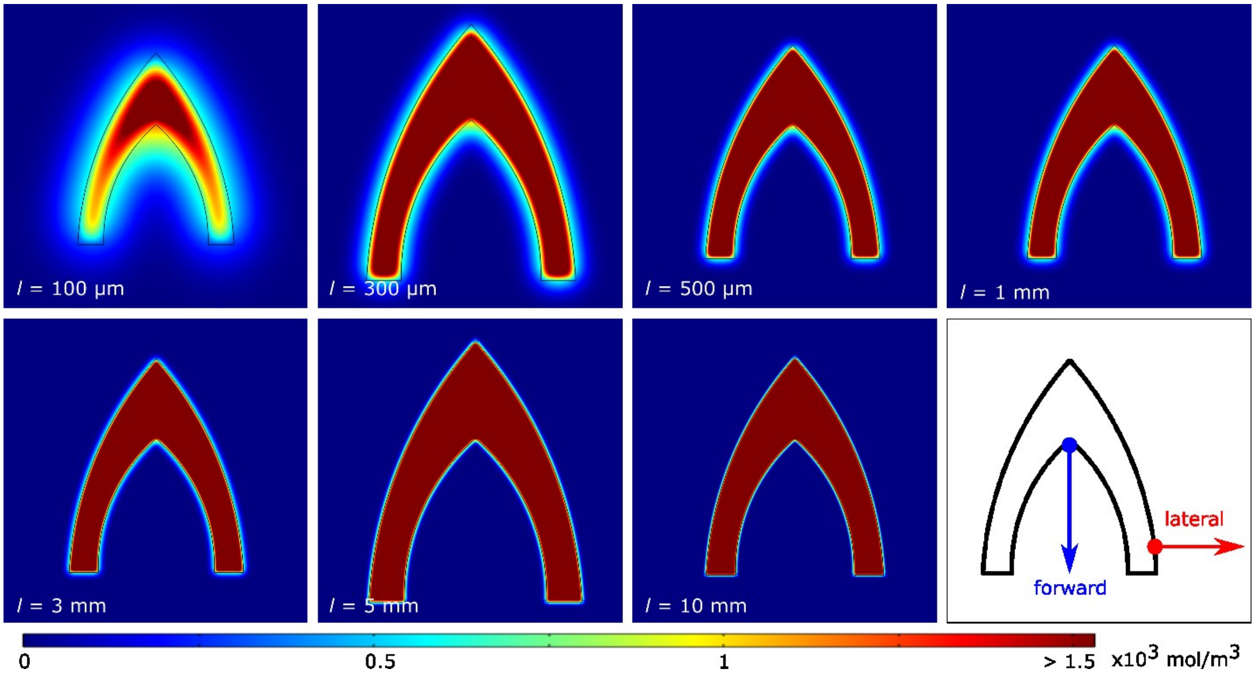

Supplementary Figure 11: Fuel release simulations in COMSOL for protein motors from *l* = 100 µm to 10 mm at 50 ms. Color scale is fuel concentration from 0 to 1.5 10^3 mol/m^3.

We simulated the release of fuel of protein motors with characteristic length scale *l* = 100 µm to 10 mm in COMSOL. The fuel release was simulated in static conditions (zero fluid speed) after 50 ms in order to evaluate the initial conditions triggering one or another propulsion mode. Protein films had an initial fuel concentration of 2 kmol/m^3, diffusion coefficient of HFIP in water $D_{medium} = 3.2 \cdot 10^{-9}$ m^2/s [11,12], and diffusion coefficient of HFIP in the protein $D_{internal} = 7.25 \cdot 10^{-10}$ m^2/s [13–15]. We can observe that, due to their design, small-scale motors (*l* = 100 µm to 1 mm) generate an anisotropic concentration profile, with an increased local concentration in the posterior cavity. Such anisotropic concentration profile results in an anisotropic surface tension gradient that generates a forward Marangoni propulsive force. On the other hand, larger motors (*l* = 3 mm to 10 mm) have an isotropic concentration profile, with an homogeneous increase in fuel concentration along the motor contact line. In this case, the preferred direction for propulsion is perpendicular to the long axis, hence lateral propulsion[16].

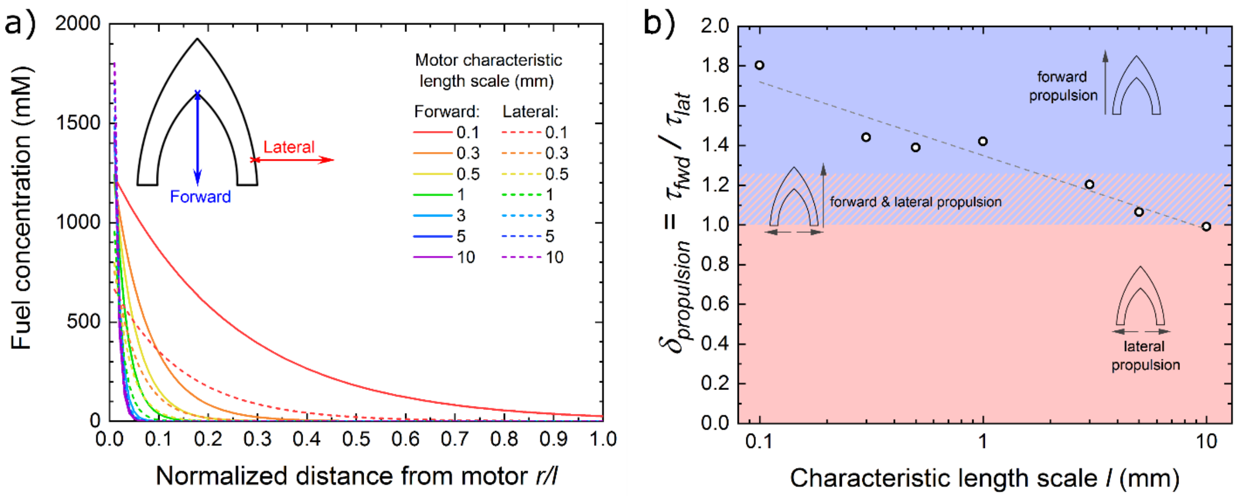

Supplementary Figure 12: a) Fuel concentration along the forward and lateral directions for protein motors from l = 100 µm to 10 mm at 50 ms as function of the normalized distance from the motor contact line r/l. **b)** Prediction of propulsion modes as function of l with $\delta_{propulsion}$ parameter.

In order to quantitatively analyze the propulsion modes, we analyzed the fuel concentration profile in two directions: along the posterior cavity (for forward propulsion) and perpendicular to the posterior leg (for lateral propulsion). In **Supplementary Figure 12a** we plotted the fuel concentration along both directions over the normalized distance from the motor contact line, r/l. We can observe that the concentration follows an exponential decay with increasing distance away from the motor contact line. While larger motors exhibit very similar sharp decay profiles for both forward and lateral directions, smaller motors exhibit different decay profiles over longer distance for forward and lateral directions. To quantify this, we calculate the decay constant τ for each exponential decay curve, and introduce the predictive parameter $\delta_{propulsion} = \tau_{fwd} / \tau_{lat}$, where τ_{fwd} and τ_{lat} are the decay constants for a given length scale motor in the forward and lateral directions respectively (**Supplementary Figure 12b**). For a $\delta_{propulsion}$ = 1 ($\tau_{fwd} = \tau_{lat}$), the fuel profiles along the forward and lateral directions are equal, creating an isotropic concentration gradient. $\delta_{propulsion}$ < 1, ($\tau_{fwd} << \tau_{lat}$), indicates the release of fuel in the lateral direction is dominant (which is not possible in the current design), and $\delta_{propulsion}$ > 1 indicates the release along the forward direction is dominant. l = 10 mm motors have $\delta_{propulsion}$ = 1, indicating an isotropic release (the leg separation is too big to create an increase in concentration in the posterior cavity). This results in lateral propulsion due to Marangoni forces perpendicular to the long axis of the motor[16]. As l decreases, the leg separation is smaller, increasing the concentration in the posterior cavity and consequently increasing $\delta_{propulsion}$. For 1.0 < $\delta_{propulsion}$ < 1.25, the anisotropy in the concentration gradient around the motor is not strong enough to guarantee continuous propulsion in the forward direction, and the motor alternates between forward and lateral propulsion. For $\delta_{propulsion}$ > 1.25, the release of fuel in the posterior cavity is dominant ($\tau_{fwd} >> \tau_{lat}$) and causes an anisotropic concentration strong enough to propel the motor forward. The prediction of propulsion modes with $\delta_{propulsion}$ agrees with our experimental observations (**Supplementary Figure 10**), and therefore we accept it as a valid design parameter for programmable locomotion of our protein self-propelled motors.

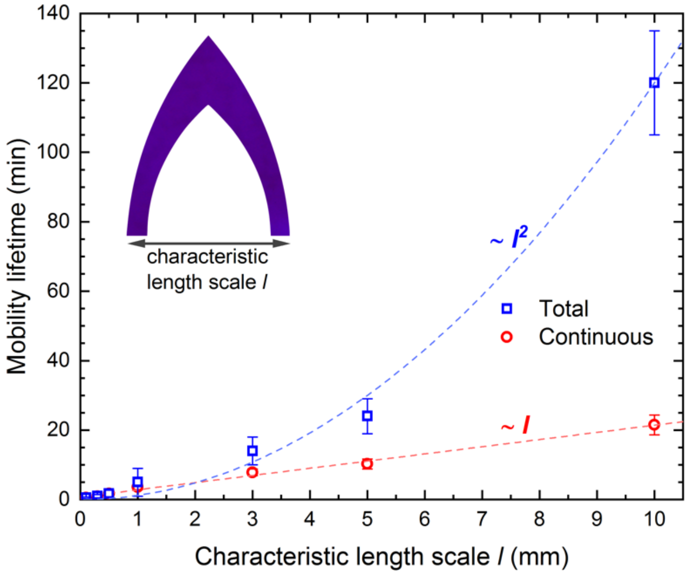

Supplementary Figure 13: **Total and continuous mobility lifetime** of protein motors across length scales. Error bars represent standard deviation, n=15.

As described in the main text, we observe two mobility regimes: an initial continuous mobility regime (where the fuel close to the interface is released) followed by an intermittent mobility regime (where the fuel close to the interface is exhausted and it is replenished by internal diffusion from the motor core). Analogous two-mobility regimes have been previously reported in other Marangoni self-propelled systems[8]. We measured the mobility lifetime of protein motors in the continuous locomotion regime and their total lifetime for all explored length scales. The mobility lifetime of the initial continuous locomotion, $\tau_{continuous}$, is related to the release of fuel close to the interface (i.e., edge of the motor body), and therefore it is expected to scale with l:

$$\tau_{continuous} \sim A_{effective}\, b_{continuous} = h\, l\, b_{continuous} \quad (1)$$

Where $A_{effective}$ is the effective propulsion area, h is motor thickness, l is characteristic length scale, and $b_{continuous}$ is an experimentally determined parameter ($b_{continuous}$ = 880 min/mm^2). Note that since the thickness is constant across length scales, $\tau_{continuous}$ scales linearly with l.

The total mobility lifetime τ_{total} (including both continuous and intermittent locomotion regimes) depends on the total amount of fuel stored in the motor, and therefore is dependent on the volume of the motor V:

$$\tau_{total} \sim V\, b_{total} = h\, l^2\, b_{total}/2 \quad (2)$$

Where b_{total} is an experimentally determined parameter (b_{total} = 960 min/mm^3). Note that since the thickness is constant across length scales, τ_{total} scales with l^2.

Supplementary Note S1. Protein motor propulsion model

We propose a propulsion model, which builds on previous work by Ayusman Sen's group[12], with the purpose of validating our experimental results and investigating the scaling laws in the protein motor design and performance. The model is based on Newton's second law. For a motor moving at terminal velocity (maximum velocity, with no acceleration), the forces acting on the motor are:

$$F_{prop} - F_{drag} = 0 \tag{3}$$

Where F_{prop} is the asymmetric surface tension force along the body contact line, and F_{drag} is the viscous drag force.

Propulsion force

The propulsion force F_{prop}, caused by the release of HFIP along the contact line and the resulting surface tension gradient, is:

$$F_{prop} = \int_{contact\ line} \sigma\, \boldsymbol{s}\, dl \ \sim \Delta\gamma\ l_{contact} \tag{4}$$

where σ is the force per length in a direction tangent to the surface, **s** is the unit vector tangent to the free surface and normal to the contact line, and dl is an incremental arclength along the contact line. If σ is constant along the contact line, the resulting F_{prop} is zero, but an asymmetric σ profile results in a net Marangoni propulsive force. For simplicity, the propulsive force can be approximated to ~ $\Delta\gamma\ l_{\mathrm{contact}}$, where $\Delta\gamma$ is an asymmetric surface tension gradient and l_{contact} is the motor body contact line. For our current motor design, $l_{\mathrm{contact}} \approx 2l$, where l is the characteristic length design parameter (see **Supplementary Figure 3**). Therefore, $F_{\mathrm{prop}} \approx 2\ \Delta\gamma\ l$.

The surface tension gradient, $\Delta\gamma = c\ k_{\mathrm{HFIP}}$, depends on the local concentration of released fuel c and on a calibration parameter k_{HFIP}. The parameter $k_{\mathrm{HFIP}} = 1.39 \cdot 10^{-4}$ N m^{2} mol^{-1} was calculated from HFIP pendant drop surface tension calibration measurements with a linear approximation at low concentrations (**Supplementary Figure 6**).

The local concentration c can be calculated as the amount of fuel released in a time interval ($J\ A\ \Delta t$) per traveled volume ($v\ A\ \Delta t$), where J_{SRT} is the diffusion flux (moles of released fuel per unit area and per second), v is the velocity, A is the area, and Δt is the time interval. $J_{\mathrm{SRT}} = 6.65 \cdot 10^{-1}$ mol m^{-2} s^{-1} was calculated from **Supplementary Figure 9** as a linear approximation for very small Δt. Therefore:

$$F_{prop} = \frac{2\, J_{SRT}\, k_{HFIP}\, l}{v} \tag{5}$$

Drag force

The drag force scales linearly with velocity (viscous drag, ~v) at low Reynolds number (Re << 1), and scales quadratically with velocity (inertial drag, ~v^2) at high Reynolds number (Re >> 1). The Reynolds number for the protein motors fall between $Re \approx 50 – 2000$ (**Supplementary Table 2**). We simulated the flow velocity field around motors with characteristic length l from 100 µm to 10 mm in COMSOL with inlet velocity v_0 (maximum speed measured experimentally, **Supplementary Table 2**) (**Supplementary Figure 14**).

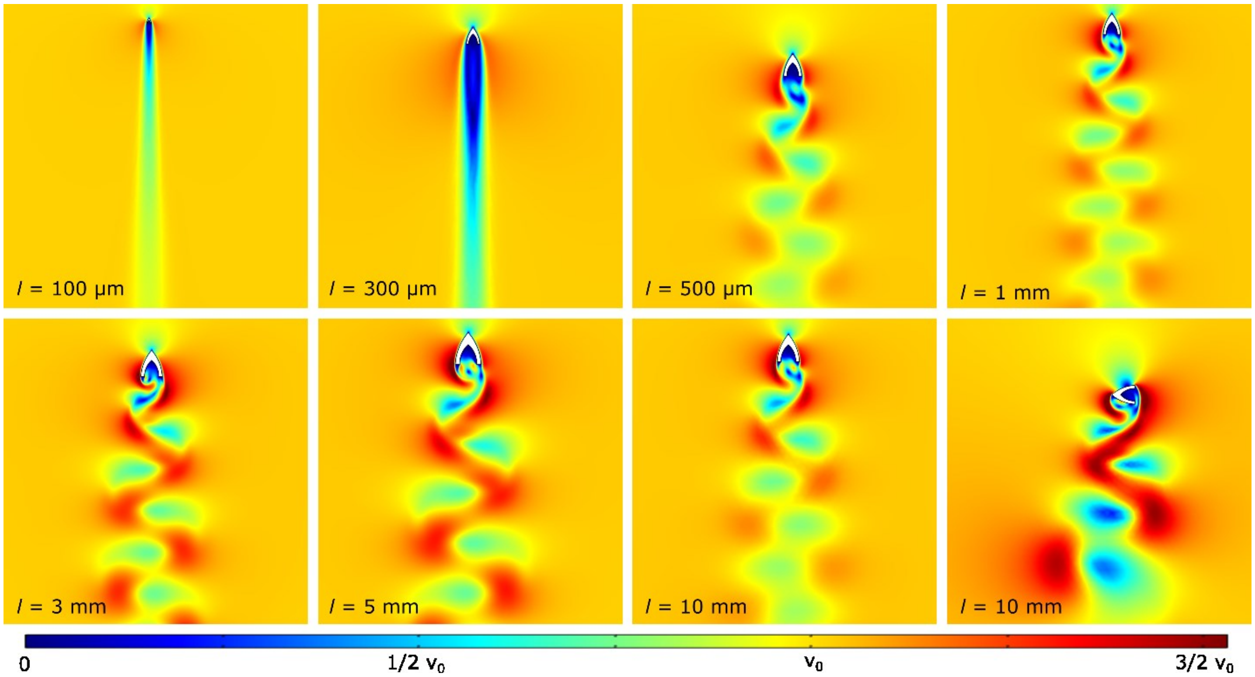

Supplementary Figure 14: COMSOL-simulated flow velocity field around motors with characteristic length l from 100 µm to 10 mm and with inlet velocity v_0 (maximum speed measured experimentally, **Supplementary Table 2**). Color scale is inlet velocity v_0.

Supplementary Table 2: experimental speed v_0 and Reynolds number for protein motors with characteristic length scale l

l (mm)	v_0 (mm/s)	Re
0.1	408.2	54
0.3	385.3	153
0.5	340.6	226
1	250.3	332
3	216.3	862
5	156.9	1042
10	148.5	1971

We observe that l = 100 μm and 300 μm motors exhibit laminar flow, while motors with characteristic length scale l = 500 μm and higher exhibit turbulent flow (with periodic vortices), indicating that our motors perform in the transition regime between laminar and weakly turbulent flow across length scales. For this reason, we cannot ignore inertial effects and calculate drag forces as:

$$F_{drag} = \frac{1}{2}\rho v^2 C_d A \tag{6}$$

Where ρ is the fluid density, v is the velocity, C_d is the drag coefficient (between 0.02 – 0.10 calculated from COMSOL simulations), and A is the area.

Calculations

If we equate $F_{prop} - F_{drag} = 0$, we can calculate the **maximum velocity v_{max}** as:

$$v_{max} = \sqrt[3]{\frac{8\, J_{SRT}\, k_{HFIP}}{\rho\, C_d\, l}} \tag{7}$$

The model is validated against experiments (**Supplementary Table 3**), showing good agreement with experimentally measured speed of protein motors across different length scales.

Supplementary Table 3. Motor speed across length scales (experimental vs. model)

l (mm)	v_{max} (mm/s) (experimental)	v_{max} (mm/s) (model)
0.1	408 ± 62	465
0.3	385 ± 43	375
0.5	341 ± 37	336
1	250 ± 30	277
3	216 ± 14	225
5	157 ± 28	195
10	148 ± 52	168

Laminar vs turbulent regime discussion

Only two motors (l = 100 μm and 300 μm) exhibited laminar flow in COMSOL simulations out of seven analyzed motors. If we consider viscous drag for small length scale motors, we can calculate the drag force from Stoke's drag equation (valid at very low Reynolds number):

$$F_{viscous\ drag} = b\, \eta\, v\, l \tag{8}$$

Where η is the fluid dynamic viscosity, v is motor velocity, l is characteristic length scale of the motor, and the b constant comes from a shape factor calculated in COMSOL for laminar flow (for these motors, $b \approx 2$). If we equate $F_{prop} - F_{viscous\ drag} = 0$, we obtain a maximum velocity of:

$$v_{\max\,(laminar)} = \sqrt{\frac{2\, J_{SRT}\, k_{HFIP}}{b\, \eta}} \tag{9}$$

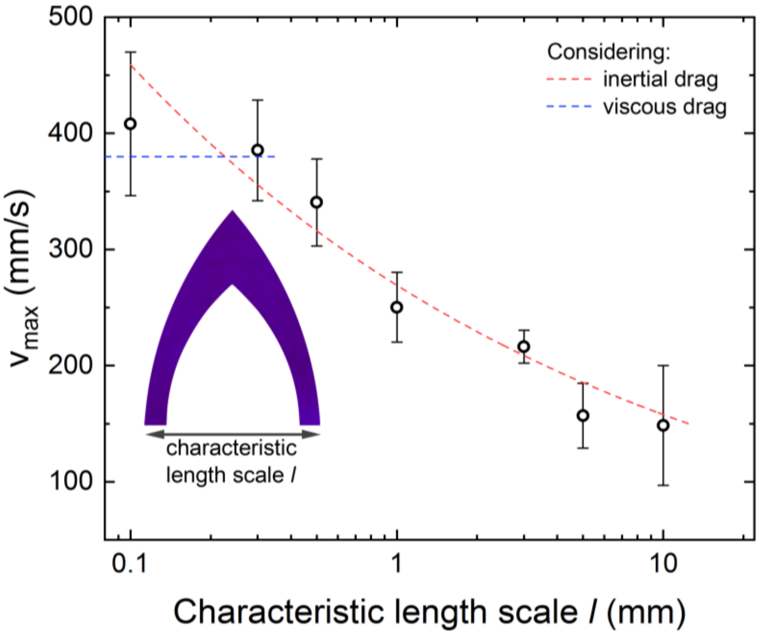

Supplementary Figure 15: motor maximum speed v_{max} as function of characteristic length scale l, considering inertial or viscous drag in calculations. Error bars represent standard deviation, n=15.

The $v_{max\ (laminar)}$ term is independent of length scale, but it is only valid for the two motors exhibiting laminar flow (l = 100 µm and 300 µm). For these two motors, we calculate a speed of $v_{max\ (laminar)}$ = 380 mm/s. We plot the calculated velocities considering inertial and viscous drag in **Supplementary Figure 15**. Calculations with inertial drag fit the experimental data well, but seem to slightly overestimate the velocity of l = 100 µm motor (due to underestimation of drag force). Nonetheless, the predicted velocity for l = 100 µm motor still falls within experimental error (standard deviation), and therefore we accept the proposed model (with inertial drag) as valid for all the explored length scales. However, if we were to explore smaller length scales, we would have to ignore inertial effects and consider viscous drag at small Reynolds numbers.

Supplementary Table 4. Motor benchmarking

Motor type	Legend	Motor efficiency $\varepsilon_{K,max}$ (μJ g^{-1})	Motor output α_{max} (mm^{-2}s^{-1})	Reference
**SRT protein motor**				
l = 10 mm	10	$5.51\cdot10^{1}$	$1.39\cdot10^{2}$	This work
l = 5 mm	5	$6.16\cdot10^{1}$	$5.89\cdot10^{2}$	"
l = 3 mm	3	$1.17\cdot10^{2}$	$2.26\cdot10^{3}$	"
l = 1 mm	1	$1.57\cdot10^{2}$	$2.35\cdot10^{4}$	"
l = 500 μm	05	$2.90\cdot10^{2}$	$1.28\cdot10^{5}$	"
l = 300 μm	03	$3.71\cdot10^{2}$	$4.01\cdot10^{5}$	"
l = 100 μm	01	$4.17\cdot10^{2}$	$3.80\cdot10^{6}$	"
**Droplet/solid fuel**				
camphor	a	$1.80\cdot10^{0}$	$7.84\cdot10^{0}$	[17]
benzoquinone	b	$2.88\cdot10^{-1}$	$3.39\cdot10^{0}$	[18]
phenanthroline	c	$2.42\cdot10^{-1}$	$2.80\cdot10^{1}$	[19]
aniline oil	d	$8.00\cdot10^{-1}$	$7.64\cdot10^{-2}$	[20]
pentanol	e	$1.25\cdot10^{0}$	$7.95\cdot10^{0}$	[21]
PECA-ethanol	f	$2.03\cdot10^{1}$	$3.00\cdot10^{-1}$	[12]
**"Boats"**				
PVDF, DMF	g	$4.46\cdot10^{0}$	$1.24\cdot10^{0}$	[22]
PDMS IPA	h	$5.16\cdot10^{1}$	$4.30\cdot10^{-2}$	[23]
SU-8 IPA	i	$5.79\cdot10^{1}$	$6.04\cdot10^{0}$	[24]
SU-8 IPA	j	$4.90\cdot10^{1}$	$2.12\cdot10^{0}$	[25]
**Polymer capsules & hydrogels**				
pNIPAAm-ethanol	k	$1.25\cdot10^{2}$	$7.35\cdot10^{1}$	[16]
polysulfone-DMF	l	$1.40\cdot10^{1}$	$8.96\cdot10^{0}$	[26]
poly(AA-co-SA), THF	m	$1.39\cdot10^{0}$	$2.00\cdot10^{0}$	[27]
agarose, camphor	n	$4.99\cdot10^{-1}$	$5.06\cdot10^{1}$	[28]
cellulose acetate, oil	o	$2.30\cdot10^{-2}$	$1.77\cdot10^{1}$	[29]
(p(VDF-co-HFP)), (EMIM-TFSI)	p	$5.78\cdot10^{1}$	$2.00\cdot10^{1}$	[30]
PA-co-PN hydrogel, SDS	q	$7.72\cdot10^{-1}$	$2.53\cdot10^{-1}$	[31]
acrylamide gel, ethanol	r	$5.00\cdot10^{-5}$	$2.50\cdot10^{-1}$	[32]
**Metal-Organic Frameworks (MOFs)**				
HKUST-1 MOF, DPA	s	$3.97\cdot10^{0}$	$4.50\cdot10^{1}$	[33]
CuJAST-1 MOF, DPA	t	$4.00\cdot10^{0}$	$6.80\cdot10^{1}$	[34]
PCN-222, DA/DMF	u	$2.20\cdot10^{1}$	$4.80\cdot10^{1}$	[8]
PCN-222, DBF	v	$5.77\cdot10^{1}$	$8.40\cdot10^{1}$	[8]
CuJAST-1 MOF, DPA	w	$3.06\cdot10^{0}$	$6.70\cdot10^{1}$	[35]

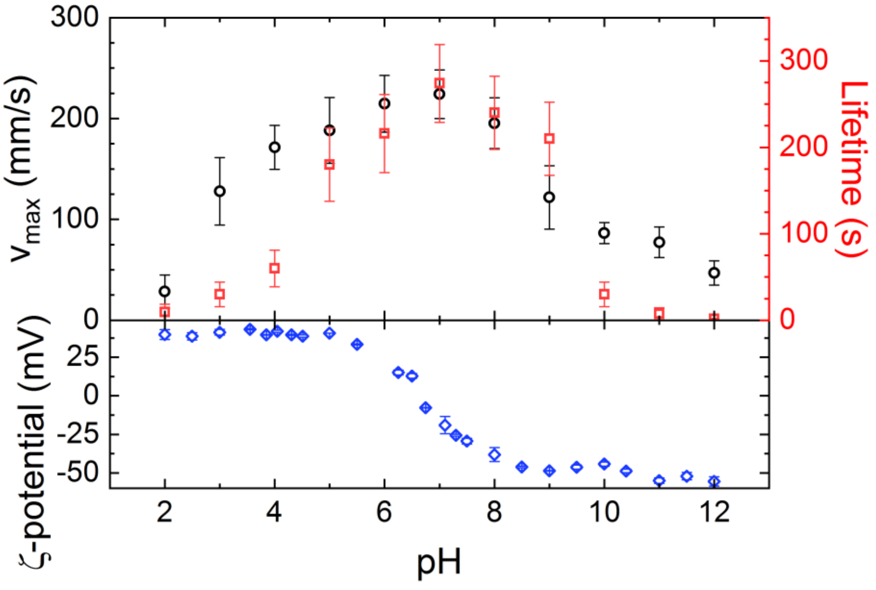

Supplementary Figure 16: Low and high pH environments reduced the mobility lifetime due to electrostatic repulsion of charged protein chains. ζ-potential data partially reproduced from Pena-Francesch et al.[36] with permission. Error bars represent standard deviation, n=15 (n=5 for ζ-potential).

SRT protein charge is dominated by histidine amino acids (11%), and therefore has an isoelectric point of ~6.7, positive charge at lower pH and negative charge at higher pH (**Supplementary Figure 16**)[36]. At pH close to physiological values (6-8), we measured high maximum speed and lifetime for l = 1 mm motors. The neutral charge favors protein aggregation into β-sheet structures in contact with water, trapping the fuel inside the protein matrix and causing a slower fuel release. At low and high pH, electrostatic repulsive forces between charged histidine blocks disrupt the formation of β-sheets, opening the protein network and thus reducing the mobility lifetime.

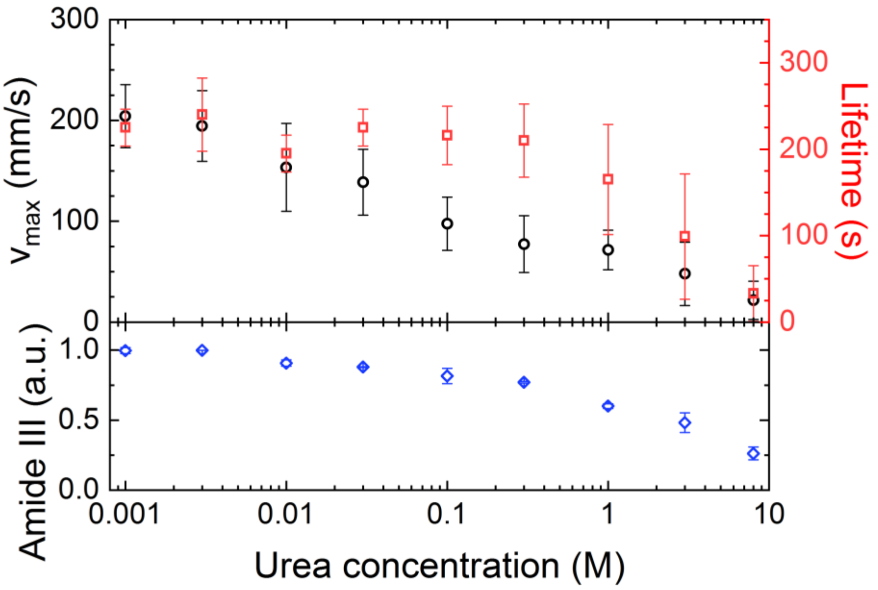

Supplementary Figure 17: Urea stimulus. High concentration of urea disrupted β-sheet nanostructures facilitating the release of fuel. Amide III intensity was measured from infrared spectroscopy in **Supplementary Figure 18**. Error bars represent standard deviation, n=15 (n=5 for Amide III intensity).

Mobility lifetime was reduced when operating the protein motors in the presence of urea, which disrupts the hydrogen bonding of β-sheets, yielding a soft, non-crosslinked, and entangled protein network[37]. With increasing concentration of urea, β-sheet nanostructures were disassembled (measured by infrared spectroscopy in **Supplementary Figure 18**), facilitating the release of fuel through a non-crosslinked protein network.

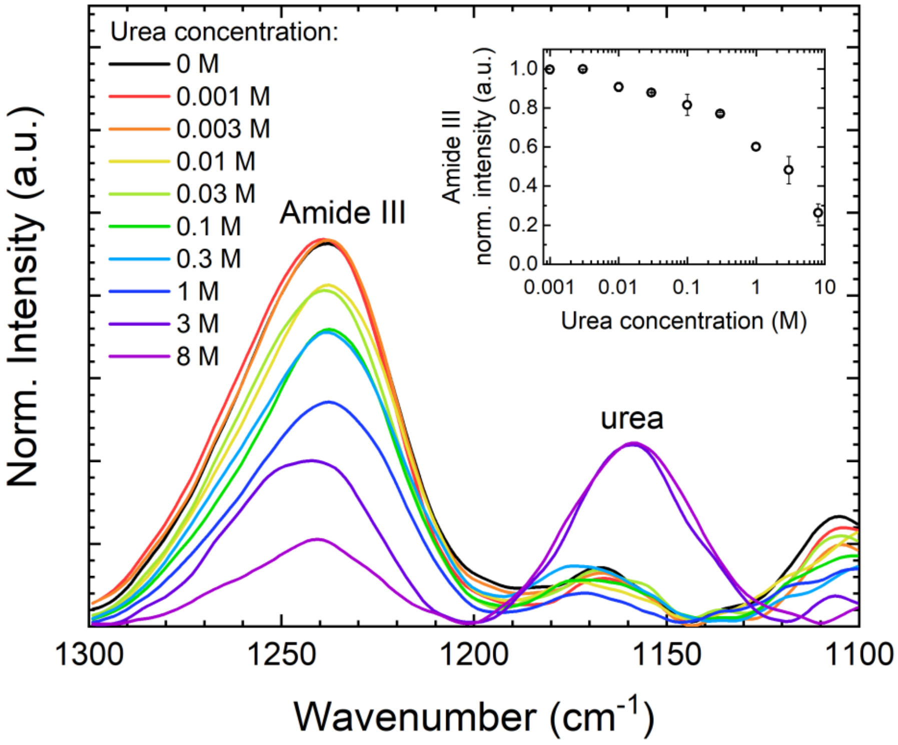

Supplementary Figure 18. Urea stimulus and protein nanostructure evolution. Urea is known to disrupt protein hydrogen-bonded nanostructures and it is commonly used in protein unfolding experiments[38–40]. Urea disrupts the β-sheet structures in SRT eliminating the physical cross-links of the protein network, observed in a decrease in intensity of Amide III (1200 – 1300 cm^{-1}) band in infrared spectroscopy[37,41].

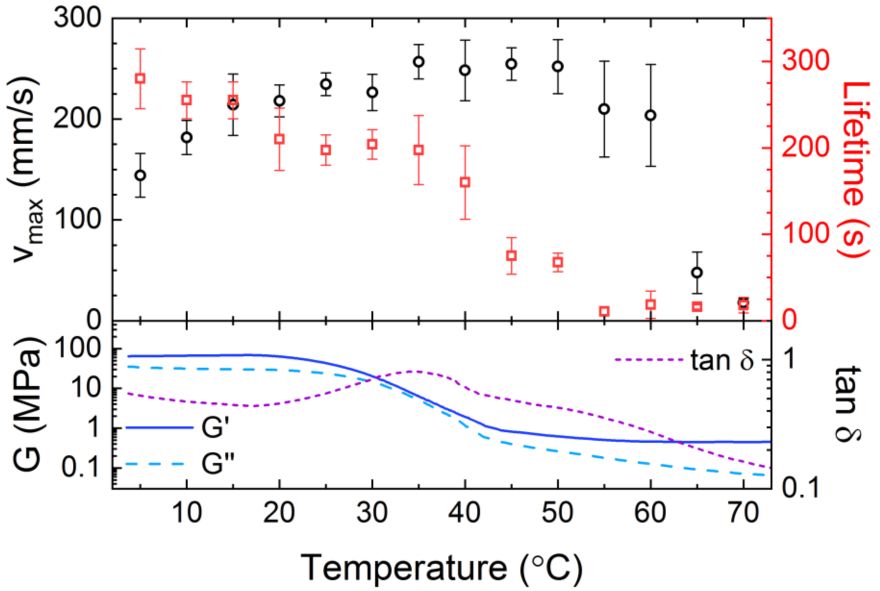

Supplementary Figure 19: Temperature stimulus. Native SRT proteins have a glass transition temperature T_g around 35 °C between a hard glassy state (rigid protein network) and a soft rubbery state (loose, flexible network)[42]. Thermomechanical analysis of protein films showed a glass transition temperature T_g at ~35 °C as a peak in *tan δ*. As temperature increases past the T_g, the fuel is easily released through the rubbery network and mobility lifetime is reduced. Error bars represent standard deviation, n=15.

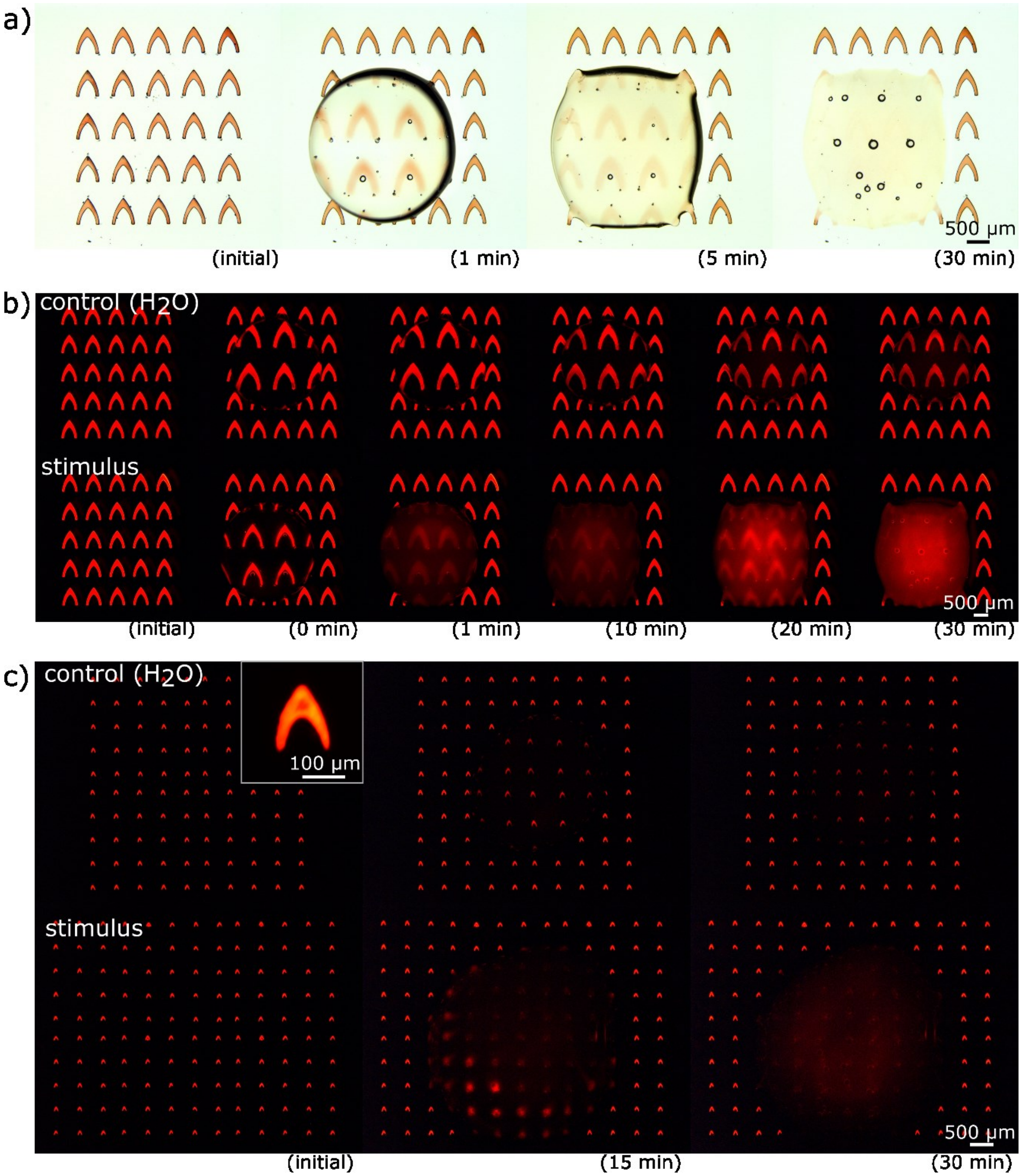

Supplementary Figure 20: Motor degradation and DOX release. a) Array of l = 500 µm DOX-loaded motors with a pH stimulus (droplet). b) Fluorescence of l = 500 µm and c) l = 100 µm DOX-loaded motors with H_2O and pH stimulus.

We demonstrated the release of doxorubicin (DOX, a commonly used chemotherapy agent) on stimuli-responsive protein motors with characteristic length scales l = 500 µm and l = 100 µm. We doped the motor solution (SRT protein + HFIP) with DOX (50 μg_{DOX} / $mg_{protein}$, 5%) and proceeded with the fabrication method previously described (film casting and laser micromachining). **Supplementary Figure 20a** showed bright-field microscopy images of a 5x5 l = 500 µm DOX-loaded motor array. A 10 µL drop of 5% diluted acetic acid was placed on top, partially wetting the array. The motors in contact with the acidic stimulus

degraded over time (disruption of the β-sheet protein cross-linking structures), releasing the encapsulated DOX. **Supplementary Figure 20b** shows fluorescence microscopy images (from natural fluorescence of DOX) of 5x5 *l* = 500 μm DOX-loaded motor arrays with H_2O and acidic stimulus droplets. Motors in contact with H_2O were stable and retained the encapsulated DOX, while motors with acidic stimulus degraded over time and released the encapsulated DOX (increasing fluorescence from the droplet). **Supplementary Figure 20c** shows the analogous experiment (degradation H_2O and acidic stimulus) with *l* = 100 μm DOX-loaded motor arrays, with similar results: *l* = 100 μm motors were stable in H_2O, but released the DOX with acidic stimulus.

Supplementary Note 2. Biocompatibility of HFIP fuel

The fuel, HFIP, is a metabolite of sevoflurane, which is an FDA-approved and widely used inhalation general anesthetic[43,44]. Approximately 5% of the sevoflurane dose is metabolized into HFIP, which is then rapidly conjugated with glucuronic acid and excreted in urine[44]. Moreover, intravenous administration of HFIP has been shown to attenuate inflammation and improve survival in murine septic peritonitis in several clinical studies[45,46]. The HFIP content of one single $l = 1$ mm protein motor is approximately 15.4 µg. Based on the reported dosage of beneficial intravenous HFIP treatments (15 µg/$g_{\text{body weight}}$), which is below the intravenous lethal dose (LD_{50} of 180 µg/$g_{\text{body weight}}$), we estimate a safe operation space of 1 $\text{motor}_{l = 1\text{ mm}}$ / $g_{\text{body weight}}$, and an upper limit of 12 $\text{motors}_{l = 1\text{ mm}}$ / $g_{\text{body weight}}$.

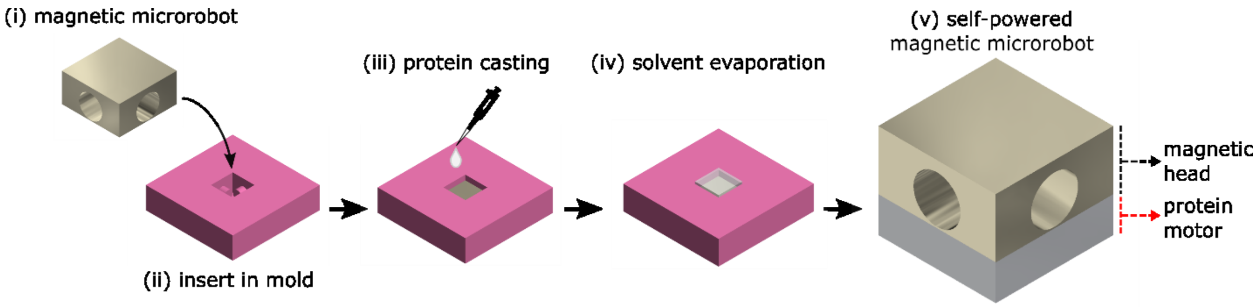

Supplementary Figure 21. Integration of modular protein motor on magnetic microrobot. (i) Magnetic microrobots were 3D-printed by two-photon lithography and coated with a 100 nm cobalt magnetic layer, as described elsewhere[47]. (ii) The microrobot was inserted in a polyurethane rubber mold, and locked inside via peg-in-hole assembly. (iii) SRT protein was cast on the top surface of the microrobot. (iv) HFIP solvent evaporated, leaving a protein film on top of the microrobot. (v) The microrobot was taken out of the mold and used for experiments. When finalized, the self-powered magnetic microrobot has two integrated components: the magnetic head (direction control by active magnetic steering) and the protein motor (integrated power source for propulsion).

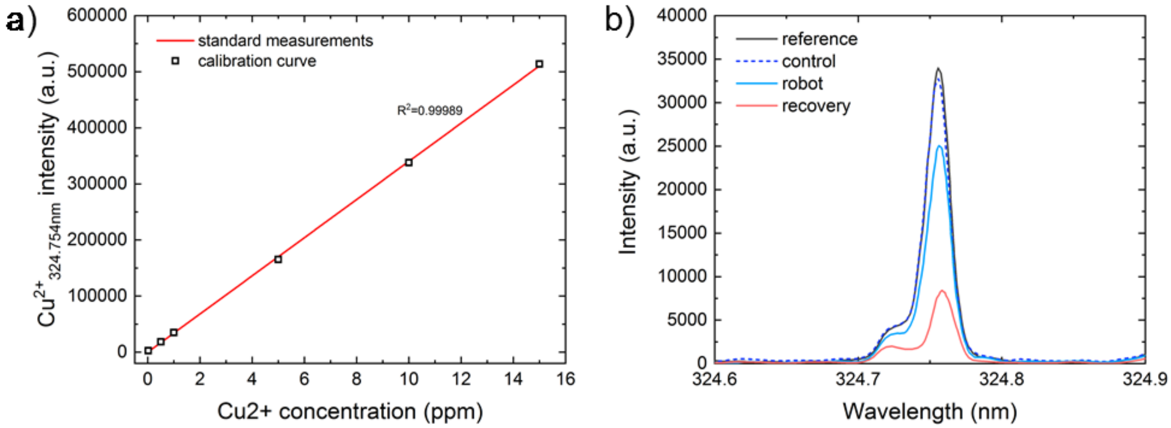

Supplementary Figure S22. Detection of Cu^{2+} ions in solution by ICP-OES. a) Cu^{2+} concentration was measured at 324.754 nm wavelength based on a 0.02 to 15 ppm calibration curve. b) ICP spectra of: 1 ppm Cu^{2+} reference, inactive protein robot control treatment, SRT protein robot treatment, and metal ion recovery after dissolving the SRT protein robot used in water treatment.

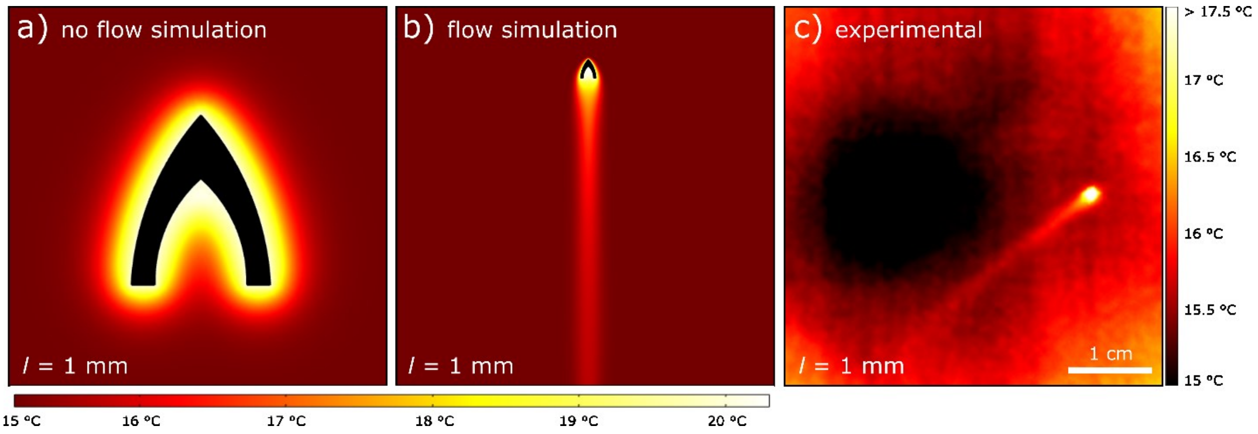

Supplementary Figure 23: **a)** Heat transfer simulation of a stationary photothermal *l* = 1 mm motor (no flow) shows an anisotropic temperature gradient with maximum temperature at the back of the motor. **b)** Heat transfer simulation of a moving photothermal *l* = 1 mm motor (flow speed 13 mm/s) shows a straight temperature trail behind the motor. Color scale is temperature from 15 to 20 °C. **c)** Infrared thermal imaging of a photothermally-propelled *l* = 1 mm motor moving in a straight line at 13 mm/s and leaving a temperature trail behind. Color scale is temperature from 15 to 17.5 °C.

Supplementary Note 3. Physical and chemical homogeneity of motors

First, the physical homogeneity of the cast protein films was examined. Defects coming from irregularities in the film (e.g., protein impurities and aggregates, microbubbles, thickness variation in areas close to the film edges) were examined by microscopy, and areas with defects were discarded from the fabrication process. Next, careful optimization of the laser micromachining process was required in order to fabricate reproducible protein motors without defects (explained in detail in **Supplementary Figure 2**). After laser micromachining, each motor was individually inspected by microscopy and those with fabrication defects (approximately 5% of the machined motors) were discarded. Therefore, only successfully fabricated protein motors without visible defects (approximately 95% yield) were used for experiments.

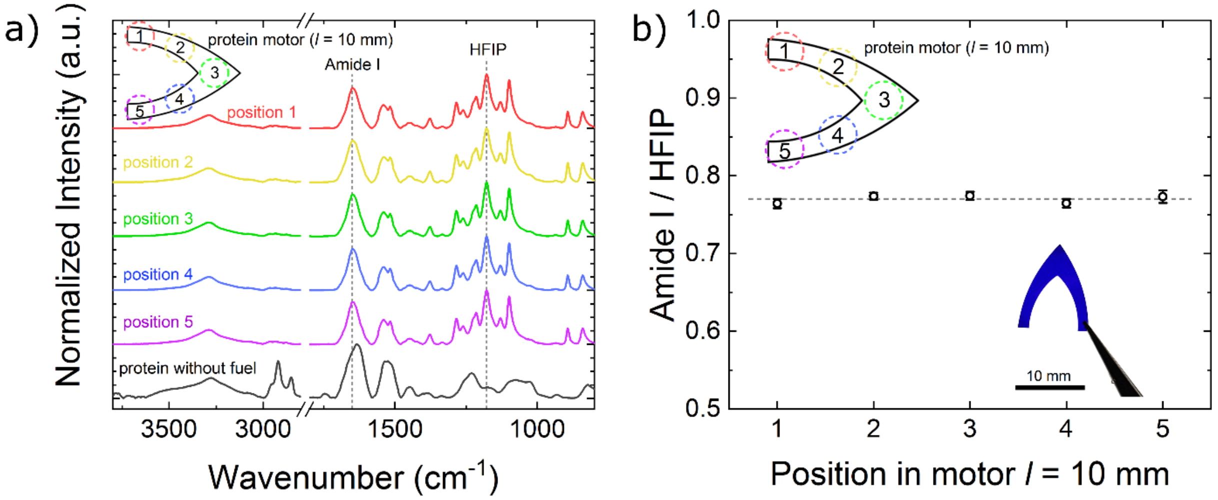

Supplementary Figure 24: analysis of chemical composition and homogeneity throughout a l = 10 mm protein motor geometry. a) FTIR spectra of l = 10 mm motor from five different locations. b) Amide I / HFIP band ratio is constant throughout the motor geometry.

Non-visible defects such as chemical composition and homogeneity of individual motors were investigated by infrared spectroscopy across the surface of motors and across motor length scales. **Supplementary Figure 24a** shows FTIR spectra of a l = 10 mm motor taken from five different locations. The five spectra are identical to each other, agreeing with previous characterization of SRT protein films. In order to quantitatively evaluate the chemical homogeneity of the motor, the ratio between the protein amide I band (1650 cm^{-1}) and the HFIP fuel band (1178 cm^{-1}) was measured (**Supplementary Figure 24b**). The amide I / HFIP ratio was constant in the different locations, indicating that the fuel composition is constant throughout the motor geometry.

In addition, we analyzed the chemical composition and homogeneity of protein motors across length scales. **Supplementary Figure 25a** shows the identical FTIR spectra of protein motors with characteristic length scale l = 10 mm to l = 100 µm. The chemical composition and homogeneity was evaluated by measuring the amide I / HFIP band ratio (**Supplementary Figure 25b**), indicating a constant fuel composition in motors across length scales.

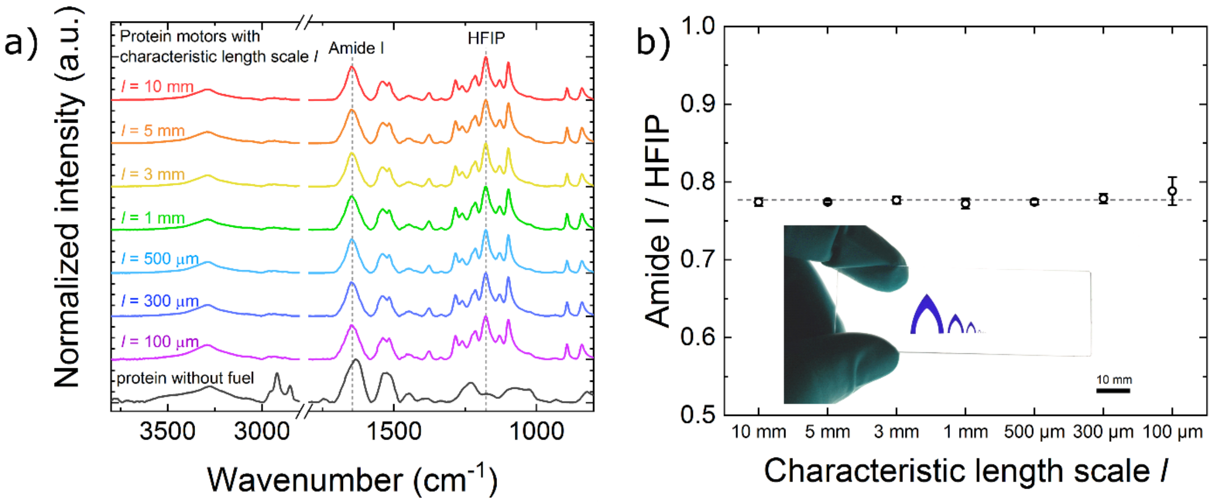

Supplementary Figure 25: analysis of chemical composition and homogeneity of protein motors across length scales. a) FTIR spectra of characteristic length scale l = 10 mm to l = 100 µm motors. b) Amide I / HFIP band ratio is constant across motor length scales.

References

1. Hu, X., Kaplan, D. & Cebe, P. Determining beta-sheet crystallinity in fibrous proteins by thermal analysis and infrared spectroscopy. *Macromolecules* **39,** 6161–6170 (2006).

2. Vazquez, G., Alvarez, E. & Navaza, J. M. Surface tension of alcohol water+ water from 20 to 50. degree. C. *J. Chem. Eng. Data* **40,** 611–614 (1995).

3. Meissner, H. P. & Michaels, A. S. Surface tensions of pure liquids and liquid mixtures. *Ind. Eng. Chem.* **41,** 2782–2787 (1949).

4. Cheong, W. J. & Carr, P. W. The surface tension of mixtures of methanol, acetonitrile, tetrahydrofuran, isopropanol, tertiary butanol and dimethyl-sulfoxide with water at 25 C. *J. Liq. Chromatogr.* **10,** 561–581 (1987).

5. N,N-Dimethylformamide (DMF)-Water Mixture Surface Tension: Datasheet from 'Dortmund Data Bank (DDB) – Thermophysical Properties Edition 2014' in SpringerMaterials (https://materials.springer.com/thermophysical/docs/msft_c72c174).

6. Markarian, S. A. & Terzyan, A. M. Surface tension and refractive index of dialkylsulfoxide+ water mixtures at several temperatures. *J. Chem. Eng. Data* **52,** 1704–1709 (2007).

7. Gente, G. & La Mesa, C. Water—Trifluoroethanol Mixtures: Some Physicochemical Properties. *J. Solution Chem.* **29,** 1159–1172 (2000).

8. Park, J. H., Lach, S., Polev, K., Granick, S. & Grzybowski, B. A. Metal–Organic Framework "Swimmers" with Energy-Efficient Autonomous Motility. *ACS Nano* **11,** 10914–10923 (2017).

9. Gil, E. S. *et al.* Mechanical improvements to reinforced porous silk scaffolds. *J. Biomed. Mater. Res. Part A* **99,** 16–28 (2011).

10. Hu, X., Cebe, P., Weiss, A. S., Omenetto, F. & Kaplan, D. L. Protein-based composite materials. *Mater. Today* **15,** 208–215 (2012).

11. Fioroni, M., Burger, K., Mark, A. E. & Roccatano, D. Model of 1, 1, 1, 3, 3, 3-hexafluoro-propan-2-ol for molecular dynamics simulations. *J. Phys. Chem. B* **105,** 10967–10975 (2001).

12. Zhang, H., Duan, W., Liu, L. & Sen, A. Depolymerization-powered autonomous motors using biocompatible fuel. *J. Am. Chem. Soc.* **135,** 15734–15737 (2013).

13. Pena-Francesch, A. *et al.* Programmable Proton Conduction in Stretchable and Self-Healing Proteins. *Chem. Mater.* **30,** 898–905 (2018).

14. Tomko, J. A. *et al.* Tunable thermal transport and reversible thermal conductivity switching in topologically networked bio-inspired materials. *Nat. Nanotechnol.* 1 (2018).

15. Marelli, B., Brenckle, M. A., Kaplan, D. L. & Omenetto, F. G. Silk fibroin as edible coating for perishable food preservation. *Sci. Rep.* **6,** 25263 (2016).

16. Bassik, N., Abebe, B. T. & Gracias, D. H. Solvent driven motion of lithographically

fabricated gels. *Langmuir* **24,** 12158–12163 (2008).

17. Kitahata, H., Hiromatsu, S., Doi, Y., Nakata, S. & Islam, M. R. Self-motion of a camphor disk coupled with convection. *Phys. Chem. Chem. Phys.* **6,** 2409–2414 (2004).

18. Suematsu, N. J., Miyahara, Y., Matsuda, Y. & Nakata, S. Self-motion of a benzoquinone disk coupled with a redox reaction. *J. Phys. Chem. C* **114,** 13340–13343 (2010).

19. Iida, K. *et al.* Experimental and theoretical studies on the self-motion of a phenanthroline disk coupled with complex formation. *Phys. Chem. Chem. Phys.* **12,** 1557–1563 (2010).

20. Chen, Y.-J., Nagamine, Y. & Yoshikawa, K. Self-propelled motion of a droplet induced by Marangoni-driven spreading. *Phys. Rev. E* **80,** 16303 (2009).

21. Nagai, K., Sumino, Y., Kitahata, H. & Yoshikawa, K. Mode selection in the spontaneous motion of an alcohol droplet. *Phys. Rev. E* **71,** 65301 (2005).

22. Zhang, L. *et al.* Marangoni effect-driven motion of miniature robots and generation of electricity on water. *Langmuir* **33,** 12609–12615 (2017).

23. Liu, X., Li, H., Qiao, L. & Luo, C. Driving mechanisms of CM-scaled PDMS boats of respective close and open reservoirs. *Microsyst. Technol.* **17,** 875–889 (2011).

24. Luo, C., Qiao, L. & Li, H. Dramatic squat and trim phenomena of mm-scaled SU-8 boats induced by Marangoni effect. *Microfluid. Nanofluidics* **9,** 573–577 (2010).

25. Qiao, L., Xiao, D., Lu, F. K. & Luo, C. Control of the radial motion of a self-propelled microboat through a side rudder. *Sensors Actuators A Phys.* **188,** 359–366 (2012).

26. Zhao, G., Seah, T. H. & Pumera, M. External-Energy-Independent Polymer Capsule Motors and Their Cooperative Behaviors. *Chem. Eur. J.* **17,** 12020–12026 (2011).

27. Gong, J. P., Matsumoto, S., Uchida, M., Isogai, N. & Osada, Y. Motion of polymer gels by spreading organic fluid on water. *J. Phys. Chem.* **100,** 11092–11097 (1996).

28. Soh, S., Bishop, K. J. M. & Grzybowski, B. A. Dynamic self-assembly in ensembles of camphor boats. *J. Phys. Chem. B* **112,** 10848–10853 (2008).

29. Liakos, I. L. *et al.* Biomimetic Locomotion on Water of a Porous Natural Polymeric Composite. *Adv. Mater. Interfaces* **3,** 1500854 (2016).

30. Furukawa, K., Teshima, T. & Ueno, Y. Self-propelled ion gel at air-water interface. *Sci. Rep.* **7,** 9323 (2017).

31. Liang, Y. *et al.* Multi-stage hydrogel rockets with stage dropping-off by thermal/light stimulation. *J. Mater. Chem. A* **6,** 16838–16843 (2018).

32. Sharma, R., Chang, S. T. & Velev, O. D. Gel-based self-propelling particles get programmed to dance. *Langmuir* **28,** 10128–10135 (2012).

33. Ikezoe, Y. *et al.* Peptide–metal organic framework swimmers that direct the motion toward chemical targets. *Nano Lett.* **15,** 4019–4023 (2015).

34. Ikezoe, Y. *et al.* Peptide Assembly-Driven Metal–Organic Framework (MOF) Motors for

Micro Electric Generators. *Adv. Mater.* **27,** 288–291 (2015).

35. Ikezoe, Y., Washino, G., Uemura, T., Kitagawa, S. & Matsui, H. Autonomous motors of a metal–organic framework powered by reorganization of self-assembled peptides at interfaces. *Nat. Mater.* **11,** 1081 (2012).

36. Pena-Francesch, A. *et al.* Pressure sensitive adhesion of an elastomeric protein complex extracted from squid ring teeth. *Adv. Funct. Mater.* **24,** 6227–6233 (2014).

37. Pena-Francesch, A. *et al.* Mechanical Properties of Tandem-Repeat Proteins Are Governed by Network Defects. *ACS Biomater. Sci. Eng.* **4,** 884–891 (2018).

38. Bennion, B. J. & Daggett, V. The molecular basis for the chemical denaturation of proteins by urea. *Proc. Natl. Acad. Sci.* **100,** 5142–5147 (2003).

39. Pace, C. N. & Scholtz, J. M. Measuring the conformational stability of a protein. *Protein Struct. A Pract. approach* **2,** 299–321 (1997).

40. Shirley, B. A. Urea and guanidine hydrochloride denaturation curves. *Protein Stab. Fold. Theory Pract.* 177–190 (1995).

41. Guerette, P. a. *et al.* Nanoconfined β-sheets mechanically reinforce the supra-biomolecular network of robust squid Sucker Ring Teeth. *ACS Nano* **8,** 7170–7179 (2014).

42. Pena-Francesch, A. *et al.* Materials Fabrication from Native and Recombinant Thermoplastic Squid Proteins. *Adv. Funct. Mater.* **24,** 7401–7409 (2014).

43. Colomer, I., Chamberlain, A. E. R., Haughey, M. B. & Donohoe, T. J. Hexafluoroisopropanol as a highly versatile solvent. *Nat. Rev. Chem.* **1,** 88 (2017).

44. US Food & Drug Administration. *ULTANE® (sevoflurane) volatile liquid for inhalation*. (2017).

45. Urner, M. *et al.* Insight into the beneficial immunomodulatory mechanism of the sevoflurane metabolite hexafluoro-2-propanol in a rat model of endotoxaemia. *Clin. Exp. Immunol.* **181,** 468–479 (2015).

46. Herrmann, I. K. *et al.* Intravenous application of a primary sevoflurane metabolite improves outcome in murine septic peritonitis: first results. *PLoS One* **8,** e72057 (2013).

47. Giltinan, J., Diller, E. & Sitti, M. Programmable assembly of heterogeneous microparts by an untethered mobile capillary microgripper. *Lab Chip* **16,** 4445–4457 (2016).
